# Doping Effect of Graphene Nanoplatelets on Electrical Insulation Properties of Polyethylene: From Macroscopic to Molecular Scale

**DOI:** 10.3390/ma9080680

**Published:** 2016-08-10

**Authors:** Ziang Jing, Changming Li, Hong Zhao, Guiling Zhang, Baozhong Han

**Affiliations:** 1 Key Laboratory of Engineering Dielectric and its Application, Ministry of Education, Harbin University of Science and Technology, Harbin 150040, China; hljjza@163.com (Z.J.); hustlichangming@163.com (C.L.); hongzhao@hrbust.edu.cn (H.Z.); hbzhlj@163.com (B.H.); 2 College of Chemical and Environmental Engineering, Harbin University of Science and Technology, Harbin 150040, China; 3 Shanghai Qifan Wire and Cable Co., Ltd., Shanghai 200008, China

**Keywords:** graphene nanoplatelets doping effect, insulation property, polyethylene

## Abstract

The doping effect of graphene nanoplatelets (GNPs) on electrical insulation properties of polyethylene (PE) was studied by combining experimental and theoretical methods. The electric conduction properties and trap characteristics were tested for pure PE and PE/GNPs composites by using a direct measurement method and a thermal stimulated current (TSC) method. It was found that doping smaller GNPs is more beneficial to decrease the conductivity of PE/GNPs. The PE/GNPs composite with smaller size GNPs mainly introduces deep energy traps, while with increasing GNPs size, besides deep energy traps, shallow energy traps are also introduced. These results were also confirmed by density functional theory (DFT) and the non-equilibrium Green’s function (NEGF) method calculations. Therefore, doping small size GNPs is favorable for trapping charge carriers and enhancing insulation ability, which is suggested as an effective strategy in exploring powerful insulation materials.

## 1. Introduction

Since the 1950s, polyethylene (PE) has been the focused insulation material due to its outstanding electrical insulation property, excellent chemical stability, good processing formation performance, as well as efficient economic benefits. PE has been widely used in power apparatus as the main insulation material (e.g., capacitors, cables) [1,2]. Currently, much effort has been devoted to enhance the electric strength and the insulation property of PE under a direct current field by using various techniques [3,4,5,6,7,8]. Particularly, using alien fillers such as ceramic oxides (e.g., Al_2_O_3_, ZnO, SiO_2_, etc.) to tailor the properties of PE has become a popular and effective strategy to develop insulation dielectrics with a specific high performance [9,10,11,12,13].

Many experimental works have found that doping of alien fillers could greatly improve the dielectric properties of PE composite and maintain high thermal endurance [14,15,16,17,18,19]. Fleming and his coworkers found that the conductivity of PE matrix composite containing 10 wt % TiO_2_ decreased by 1–3 orders relative to undoped PE [20]. Yang et al. reported that the direct current conductivity of PE/SiO_2_ decreased by 1–2 orders relative to pure PE [21]. Tian et al. stated that doping ZnO could introduce large amounts of deep trapping states and result in a decreasing conduction current of the PE/ZnO composite [22]. Ishimoto et al. found that the conductivity of PE/MgO composite was decreased compared with pure PE, irrespective of the size of MgO [23]. In prior works, PE/graphene with low content of graphene was generally used for electromagnetic shielding and anti-static electricity materials [24,25,26]. In this work, we found that graphene nanoplatelets (GNPs) are another good candidate for doping PE which can evidently lower the conductivity of the PE/GNPs composite. To the best of our knowledge, this is the first contribution regarding the doping effect of GNPs on the insulation properties of PE/GNPs composites.

Some theoretical works have been devoted to investigating the micro process and physical nature of the electrical phenomenon of the PE matrix composite. Montanari et al. calculated the electronic structure of PE and suggested a 6.0 eV forbidden band gap [27]. Righi et al. studied the electronic structure of the PE surface by considering an orthorhombic crystalline PE slab and the electron affinity was calculated to be −0.10 and −0.17 eV for the (001) and (110) surface, respectively [28]. Huzayyin and his coworkers used density functional theory (DFT) to study the effect of various chemical impurities in PE on the electronic structures, trap depths, electron densities, and inter-chain interactions. They stated that both shallow traps and deep traps could be introduced into PE by impurities [29]. Based on DFT and ab initio calculations, Meunier et al. validated that physical and chemical defects could both trap electrons [30,31,32]. To the best of our knowledge, no theoretical works have been related to GNPs doping effect on the dielectric property of PE.

In this work, the doping effect of GNPs on the dielectric property of PE was investigated by combining experimental and theoretical methods. The conduction properties and trap characteristics were tested for pure PE and PE/GNPs composites firstly by using a direct measurement method and a thermal stimulated current (TSC) method. Then, the trap-limited band conduction properties of PE and PE/GNPs composites were studied using DFT and Non-Equilibrium Green Formula (NEGF) methods. The theoretical analysis qualitatively correlates well with the results from the experiment.

## 2. Experimental Works

### 2.1. Samples Preparation

Low density PE (LDPE) was used in this study, which was produced by SINOPEC Beijing Yanshan Petrochemical Co., Ltd., Beijing, China. The GNPs were manufactured by XG Sciences, Inc., Lansing, MI, USA. Two kinds of GNPs with different sizes (denoted as GNP(1) and GNP(2), respectively) were selected for fabricating PE/GNPs composites. The maximum diameters of flake plane of GNP(1) and GNP(2) were about 1.0 and 0.65 μm, respectively, which were obtained through a proprietary manufacturing process by the manufacturer [33]. The content of both kinds of GNPs was 1.0 wt % and the thickness was both 30–40 nm. Therefore, GNP(2) has more flakes than GNP(1) at the same quality. The PE/GNPs composite was prepared by a melt blending method using a torque rheometer. The LDPE was first melted in the torque rheometer for 5 min at 383 K with the speed of the rotors at 50 rpm, and then the GNP(1) or GNP(2) was filled into the LDPE. The GNPs and PE were mixed for 20 min at 393 K with the speed of the rotors at 70 rpm. After that, both the pure PE and the two kinds of PE/GNPs composites were pressed into thin films under a plate vulcanizer at 383 K, with thicknesses of either 80 or 200 μm, the thicker films being used in the conduction characteristic test and the thinner films in the TSC test. All samples were evaporated with an Al electrode on both sides with a diameter of 25 mm.

### 2.2. Conduction Characteristic Test 

**Figure 1:**
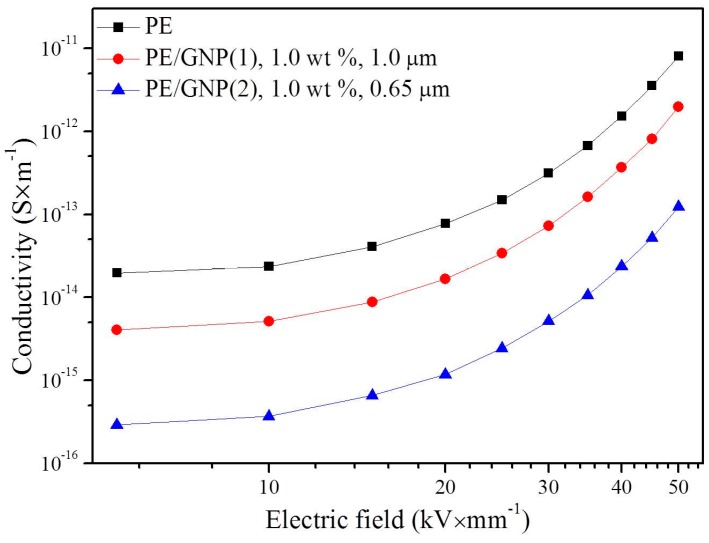
*Υ*-*E* characteristic curves of pure PE, PE/GNP(1), 1.0 wt %, 1.0 μm composite, and PE/GNP(2), 1.0 wt %, 0.65 μm composite (PE and GNP refer to polyethylene and graphene nanoplatelet, respectively).

Based on the direct measurement method, the conduction properties of pure PE, PE/GNP(1) composite, and PE/GNP(2) composite were tested. Quasi steady state current was obtained by the electrometer, Keithley6517B. The measurement was carried out at room temperature. Step voltages from 1000 to 10,000 V were applied on the samples with 200 μm thickness. The interval of the step voltages was 1000 V and the sampling time was 600 s. For ensuring accuracy of the experimental results, the measurement was repeated four times under every testing voltage. The final result was the average value of the four measurements at every testing voltage. The obtained conductivity-electric field (*Υ*-*E*) characteristic curves are shown in Figure 1.

### 2.3. Electron Trap Characteristic Test

**Figure 2:**
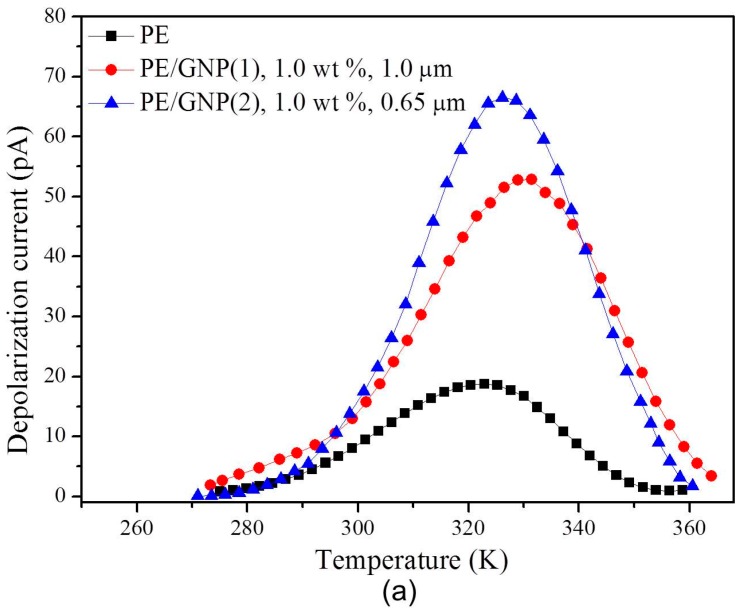

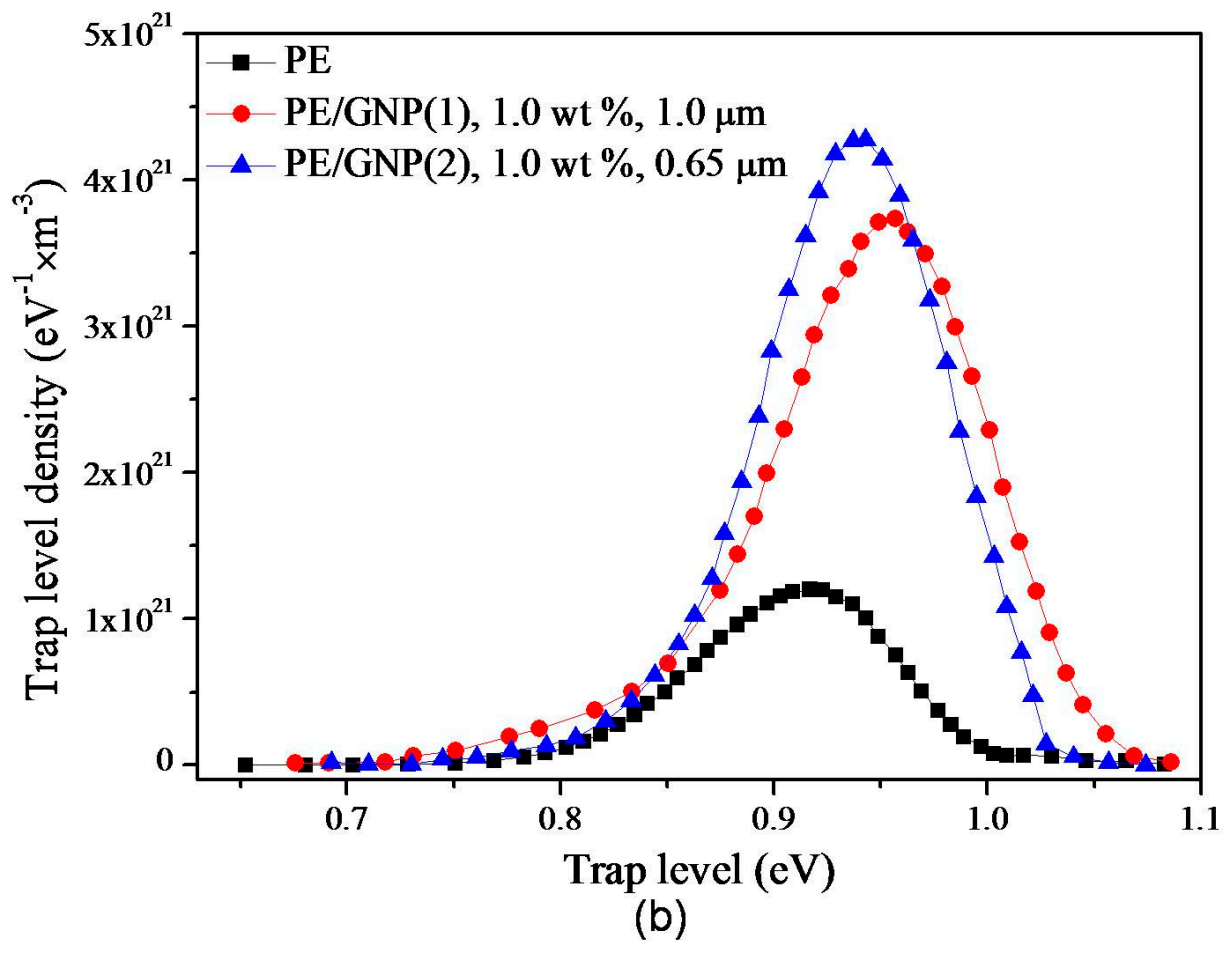
(**a**) Depolarization current versus temperature of pure PE, PE/GNP(1), 1.0 wt %, 1.0 μm composite, and PE/GNP(2), 1.0 wt %, 0.65 μm composite; (**b**) Trap level distribution of pure PE, PE/GNP(1), 1.0 wt %, 1.0 μm composite, and PE/GNP(2), 1.0 wt %, 0.65 μm composite.

The electron trap characteristic was tested based on the TSC method. The TSC method is a significant method to study important parameters of electrets such as thermal charge mobility and trapped charge density, etc. For example, using the TSC method, He et al. obtained the trap distribution of PE/MgO, Tian et al. discussed the trap property of PE/ZnO, and Han et al. investigated the trap distribution of PE/zeolite [34,35,36]. In this work, the sample was firstly charged under dc electric field (40 kV/mm) at 323 K. After charging 30 min, the sample was rapidly cooled to 270 K, and then shorted until the current decayed to below 2 pA for eliminating the influence of stray current and interfacial charge. Then, the sample was heated to 370 K at aspeed of 2 K/min. The change of depolarization current versus the temperature was recorded by Labview software and the result is shown in Figure 2a.

### 2.4. Results and Discussion

Figure 1 shows the *Υ*-*E* characteristic curves of pure PE and GNPs/PE composites. Clearly, adding GNPs into PE could markedly reduce the conductivity on comparing with pure PE. The conductivity of PE/GNP(1) composite is about one order of magnitude lower than that of pure PE. Particularly, PE/GNP(2) composite exhibits lower conductivity than PE/GNP(1) composite. This means that doping smaller GNPs flack is more beneficial in decreasing the conductivity of PE/GNPs. Such a case is also confirmed by theoretical prediction as shown in the following section.

The depolarization currents versus temperature for the thermal activation for pure PE, PE/GNP(1), and PE/GNP(2) are plotted in Figure 2a. The voltage was applied for 30 min so that the space charge could be well accumulated in the PE or PE/GNPs composites. The depolarization current and the temperature are important parameters in investigating charge carrier trap characteristics. Generally, the deeper the trap, the higher is the trap energy, that is, a higher temperature is needed for detrapping of the trapped charge. If ignoring retrapping of the detrapped charge and the recombination of different polar carriers, all the detrapping charges contribute to the depolarization current. The relationship between the depolarization current *I*, the trap energy *E*, and the temperature *T* are given as Equation (1) [37].

(1) $$J(T)\;=\frac{I(T)}{S}\;=\frac{el^{2}}{2d}\int_{E_{v}}^{E_{c}}f_{0}\left(E\right)N_{t}\left(E\right)e_{n}e^{-\frac{1}{\beta }\int_{T_{0}}^{T}e_{n}dT}dE$$

where

(2) $$e_{n}=v\exp(-\frac{E_{t}}{kT})$$

*J*(*T*) is the current density, *S* is the area of the electrode, *e* is electronic charge quantity, *f*_0_ is an equation referring to the initial occupancy of a trap level, *N_t_* is the trap level density of the localized states, *E* is the trap energy, *e_n_* is the rate of emission of electrons from a level at energy *E* and temperature *T*, *ν* is the escape frequency of trapped electrons, *k* is the Boltzmann constant, *d* is the thickness of the film, *l* is the penetration depth of the injected electrons, and *β* is the heating rate.

According to the numerical calculation method addressed by Tian et al. [38], Equation (1) can be simplified as Equation (3).

(3) $$\begin{array}{ll} J(t) & =\frac{el^{2}}{2d}f_{0}\left(E_{m}\right)N_{t}\left(E_{m}\right)\frac{dE_{m}}{dt}\\ & =\frac{el^{2}}{2d}f_{0}\left(E_{m}\right)N_{t}\left(E_{m}\right)\frac{kT}{t} \end{array}$$

where

(4) $$t=\frac{T-T_{0}}{\beta }$$

(5) $$E_{m}=kT\ln[\frac{v(T-T_{0})}{\beta }]$$

*E*_m_ is also defined as the demarcation energy [38].

By combining Equations (3) and (4), Equation (3) can be converted to Equation (6).

(6) $$f_{0}N_{t}(E_{m})=\frac{2d(T-T_{0})}{el^{2}\beta kT}J(T)$$

Assuming all the traps were fully filled and $f_{0}=1$, by combining Equations (5) and (6), the trap level density of pure PE, PE/GNP(1), and PE/GNP(2) can be obtained as plotted in Figure 2b. The trap energy levels for both PE and PE/GNPs composites are in the range of 0.70–1.10 eV. The trap level density peak of pure PE is around 0.92 eV, consistent with the result of 0.92 eV obtained by Ieda et al. [39]. Evidently, the peak of the trap level density shifts to higher energy level after introducing the GNPs dopants. The density peaks of PE/GNP(1) composite and PE/GNP(2) were around 0.95 eV and 0.93 eV, respectively. At a lower energy level (<0.86 eV), the trap density of PE/GNP(1) is larger than PE/GNP(2). And at a higher energy level (>0.98 eV), the trap density of PE/GNP(1) is larger than PE/GNP(2). This means that PE/GNP(2) with a smaller size GNP(2) mainly introduces deep energy traps while PE/GNP(1) with a larger size GNP(1) introduces not only deep energy traps but also shallow energy traps. The effectiveness of the deep traps and shallow traps on the process of carrier transport is different [40]. The shallow trap states are in thermal equilibrium with valence band (VB) or conduction band (CB): transport can occur by thermal activation of carriers to VB or CB, leading to the concept of conduction through multiple trapping/detrapping steps, which is beneficial to enhancing the charge transport. However, the deep trap states are far away from VB or CB, and this is thought to control the space charge and hinder the charge transport. Therefore, small size GNPs are beneficial in trapping charge carriers and enhancing insulation ability. In order to further illustrate the energy level changes with the introduction of GNPs, the volume average trap densities of the samples can be estimated through the integral of the trap level density curves. The volume average trap densities of pure PE, PE/GNP(1) composite, and PE/GNP(2) composite, are 1.38 × 10^20^ m^−3^, 4.54 × 10^20^ m^−3^, and 4.98 × 10^20^ m^−3^, respectively. PE/GNP(2) has higher volume trap densities than PE/GNP(1) owing to the fact that PE/GNP(1) has a larger amount of molecular segments than PE/GNP(2).

## 3. Theoretical Works

**Figure 3:**
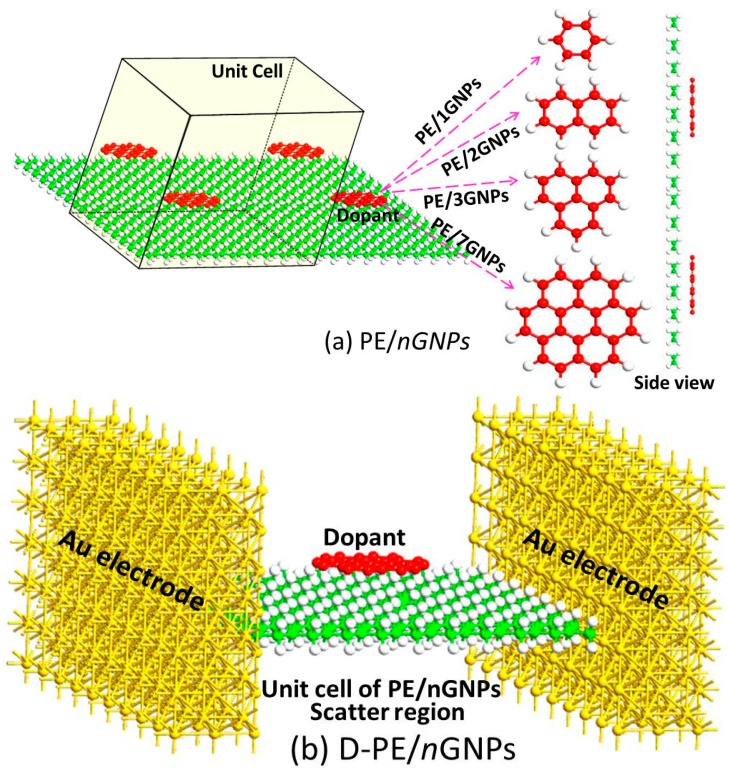
(**a**) Optimized structures of PE/*n*GNPs (*n* = 1, 2, 3, 7). The unit cell for calculations is also given; (**b**) Two-probe devices of PE/*n*GNPs (*n* = 1, 2, 3, 7) as one supercell were sandwiched between two Au (100)−(7 × 9) electrodes, five layers were used for the left and right Au electrodes.

In order to qualitatively investigate the doping effect of GNPs on the dielectric property of PE/GNPs composite, we constructed calculation models by assuming graphene flacks were added into PE, as shown in Figure 3. The electronic structures and transport properties of PE and PE/GNPs were analyzed by combining the calculated results from the DFT and NEGF methods.

### 3.1. Electronic Structure Calculation

For computing electronic structures, the infinite system of pure PE and PE/GNPs was modeled using a periodic condition in the plane of YZ. Four models were chosen here, denoted as PE/*n*GNPs, *n* = 1, 2, 3, and 7, meaning one-ring graphene, two-ring graphene, three-ring graphene, and seven-ring graphene were introduced into one unit cell of PE, respectively (Figure 3a). Each unit cell contained eight PE chains and each PE chain consisted of twenty C atoms. These eight PE chains were parallel arrayed along the Y-axis direction. The longitudinal repeat direction of the PE chain was along the Z-axis. The graphene plane was also in the YZ plane and superposed upon the 8 PE plane. All the periodic systems were fully optimized until the maximum absolute force was less than 0.02 eV/Å. Computations for the infinitely long systems were performed using an ab initio code package, Atomistix ToolKit (ATK), which is based on combination of DFT and NEGF methods [41,42,43,44]. A generalized gradient approximation (GGA) within the Perdew-Burke-Ernzerhof (PBE) formalism was employed to describe the exchange correlations between electrons. A double-*ζ* basis functional with polarization (DZP) was used for all atoms. The k-point was set as (1 × 50 × 50) in the Brillouin zone (*x*, *y*, *z* directions, respectively).

### 3.2. Electronic Structure

**Figure 4:**
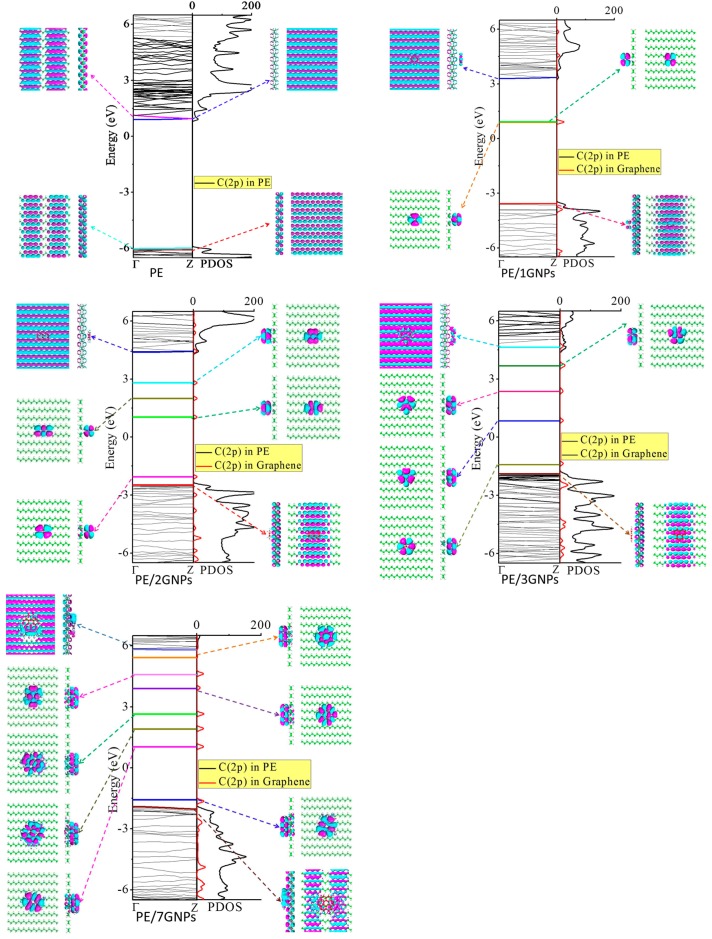
Calculated band structures and PDOS of PE, PE/1GNPs, PE/2GNPs, PE/3GNPs, and PE/7GNPs and the Kohn-Sham orbitals corresponding to the energy levels (highlighted in color lines) near *E*_f_ at the Γ point. The iso-surface value is 0.005 eV.

Figure 4 plots the band structures and average projected density of states (PDOS) of PE, PE/1GNPs, PE/2GNPs, PE/3GNPs, and PE/7GNPs, as well as the Kohn-Sham orbitals near the Fermi level (*E*_f_). The distance between the PE and the graphene is around 3.0 Å, suggesting a Van der Waal’s interaction. For pure PE, the forbidden band spans a large band gap of about 6.2 eV, indicating typical insulation character. This is in agreement with previous experimental observations and DFT calculations [27]. In pure PE, the Kohn-Sham orbitals of VB and CB seem to cover the whole of the main chain, and thereby, the electron and hole in CB and VB are freely mobile and show no localized level. The PE VB state spreads mainly along the longitation chains, while the CB state extends not only to the longitation chains, but also overlaps between the inter-chains [40]. Such inter-chain character for CB and the intra-chain character for VB were also confirmed by Serra [45]. Therefore, carrier transport in PE may proceed via two transport pathways: one is named direct intra-chain mechanism whose carriers can transport along a PE chain; another is named indirect inter-chain mechanism whose carriers can transport by hopping to a neighboring PE chain.

When graphene pieces are introduced into PE, localized impurity bands originating from the graphene segment appear in the forbidden band region. As the size of the graphene plane increases, the amount of the impurity states in the forbidden band increases. Most of them are localized above the *E*_f_, serving as electron trap states. Based on the difference between the trap energy level and *E*_f_, the traps are divided into deep traps and shallow traps. The trap depth for the electron can be evaluated as the difference between the unoccupied impurity band nearest CB and CB. The trap depth for the hole is also calculated as the difference between the occupied impurity band nearest VB and the VB. The calculated electron trap depths for PE/1GNPs, PE/2GNPs, PE/3GNPs, and PE/7GNPs are 2.2, 1.7, 0.6, and 0.3 eV, respectively. The calculated hole trap depths for PE/nGNPs are of about 0.0–0.4 eV, acting as shallow traps. For PE/1GNPs, two degenerate graphene bands locate just above the *E*_f_ with a large trap depth, serving as deep trap states for localized electrons. On enlarging the graphene segment, more shallow trap and deep trap states are introduced below the CB. Especially, in PE/7GNPs, a series of quasi-continuous graphene bands are formed in the forbidden band region of PE. On the other hand, the hole trap state is also introduced above the VB in PE/2GNPs, PE/3GNPs, and PE/7GNPs. Moreover, adding graphene segments also induces upshift of VB, and this upshifting becomes larger with the increasing size of graphene. This means that adding a large graphene segment is prone to electron ionization from PE VB into the trap state. In other words, in the PE/GNPs composite with large graphene segments, electrons could easily fall into graphene species, transfer within the graphene itself, and then be detrapped to the CB of PE. The trap depths for the electron are decreased with increasing size of the graphene segment. Therefore, introducing graphene into PE has twofold opposite effects. One is constructive to the insulator property of PE, especially for small graphene segments; the other is deconstructive to the insulator property of PE, especially for large graphene segments.

### 3.3. Transport Property Calculation 

For computing transport properties, we carved out one unit cell of PE, PE/1GNPs, PE/2GNPs, PE/3GNPs, and PE/7GNPs as the central scatter region based on the optimized periodic structures to be sandwiched between two Au electrodes (Figure 3b). The unit cell was long enough (~25 Å) to neglect the interaction between the left and right electrodes. The semi-infinite Au electrodes were modeled by two Au (100)−(7 × 9) surfaces, and five layers were used for the left and right side. Calculations were carried out by changing the applied bias in the step of 0.2 V in the range of −1.0~1.0 V. The five two-probe devices were denoted as D-PE, D-PE/1GNPs, D-PE/2GNPs, D-PE/3GNPs, and D-PE/7GNPs, respectively. Computations were performed using ATK package. A generalized gradient approximation (GGA) within the Perdew-Burke-Ernzerhof (PBE) formalism is employed to describe the exchange correlations between electrons. A double-æ basis functional with polarization (DZP) was used for all atoms. The k-point was set as (1 × 50 × 50) in Brillouin zone (*x*, *y*, *z* directions, respectively).

### 3.4. Transport Property

**Figure 5:**
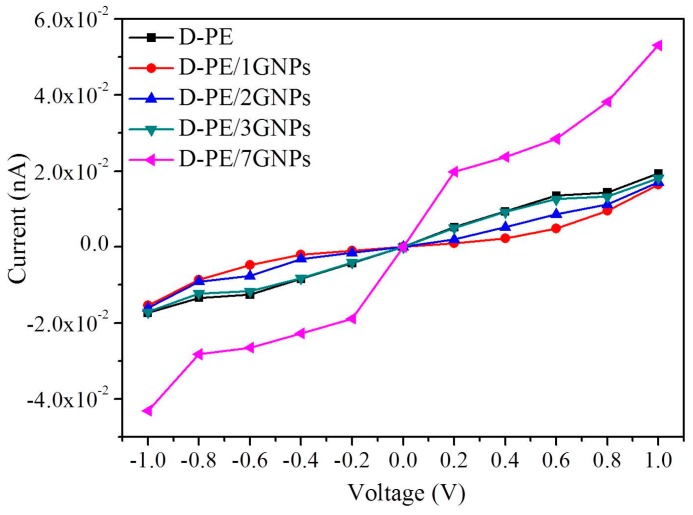
Computed *I*-*V* curves of D-PE, D-PE/1GNPs, D-PE/2GNPs, D-PE/3GNPs, and D-PE/7GNPs two-probe devices.

**Figure 6:**
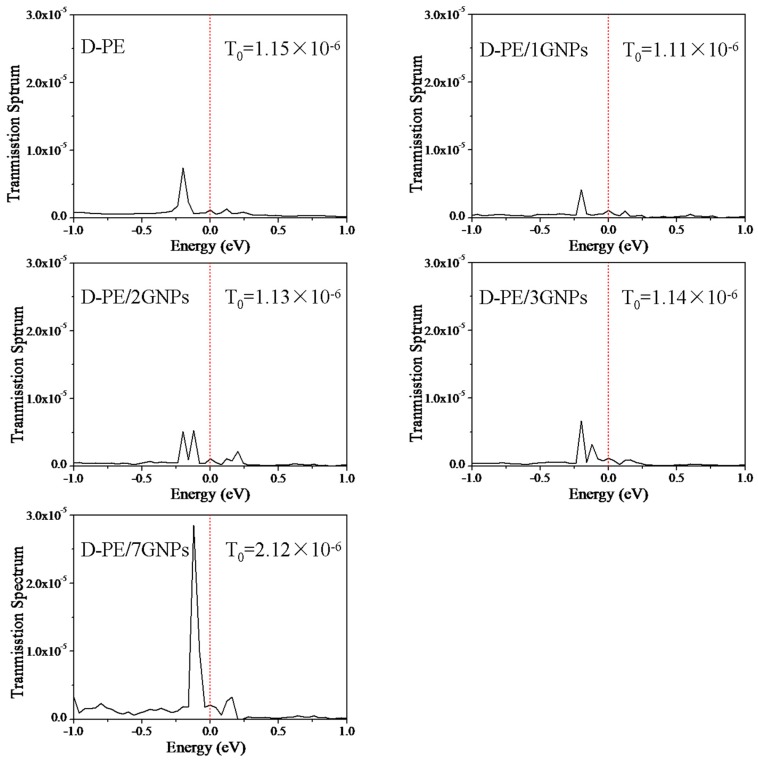
Transmission spectra of D-PE, D-PE/1GNPs, D-PE/2GNPs, D-PE/3GNPs, and D-PE/7GNPs two-probe devices at 0.0 V bias voltages.

The calculated *I*-*V* curves are shown in Figure 5. From Figure 5, the conduction current in D-PE/1GNPs and D-PE/2GNPs is decreased compared to the pure PE under certain external bias voltages. However, with increasing graphene flack size, the conduction current of the doped PE matrix tends to increase. When the seven-ring graphene flacks are filled into PE, the conduction current is even larger than the pure PE. Usually, the conduction current is relevant to the transmission peak at the *E*_f_ under free bias. Figure 6 gives the transmission spectrum (TS) at 0.0 V. Clearly, the values of TS at *E*_f_ (T_0_) follow the sequence of D-PE/1GNPs < D-PE/2GNPs < D-PE/3GNPs ≈ D-PE < D-PE/7GNPs. This result is consistent with the *I*-*V* character in Figure 5. Two reasons may contribute to the high conduction current of D-PE/7GNPs. One is that trapped carries could be easily detrapped from the shallow trap levels of graphene to the PE. Another is that the seven-ring graphene flack is large enough in the scatter region to serve as a transport channel directly.

From the theoretical conclusion based on the PE/GNPs composite, we can deduce that adding appreciable amount of GNPs of a small size into PE is favorable for enhancing the insulation property of PE. This is in agreement with the experimental result as mentioned above.

## 4. Conclusions

The doping effect of GNPs on the dielectric properties of PE was studied by combining experimental and theoretical methods. The conduction properties and trap characteristics were tested for pure PE and PE/GNPs composite by using a direct measurement method and the TSC method. It was found that doping smaller GNPs is more beneficial to decrease the conductivity of PE. The PE/GNPs composite with smaller size GNPs mainly introduces deep energy traps, while with increasing GNPs size, besides deep energy traps, shallow energy traps are also introduced. These results are also confirmed from the band structures and *I*-*V* curves of a series of PE/GNPs composites obtained from DFT and NEGF calculations. Therefore, doping small size GNPs is beneficial to trapping charge carriers and enhancing insulation ability, which is suggested as an effective strategy to explore powerful insulation materials.
